# Inflammatory and Complement Biomarkers of Cognitive Decline in Astrocyte‐Derived Extracellular Vesicles from the Vietnam Era Twin Study of Aging

**DOI:** 10.1002/alz70856_107567

**Published:** 2026-01-09

**Authors:** Michaela Cullum‐Doyle, Matthew S. Panizzon, Victoria Risbrough, Jeremy A. Elman, Carol E. Franz, William S. Kremen, Robert A. Rissman

**Affiliations:** ^1^ University of California San Diego, La Jolla, CA, USA; ^2^ Alzheimer's Therapeutic Research Institute, University of Southern California, San Diego, CA, USA; ^3^ Veterans Affairs San Diego Healthcare System, La Jolla, CA, USA

## Abstract

**Background:**

Traumatic brain injury (TBI) is a significant risk factor for Alzheimer's disease (AD) and cognitive decline, yet biomarkers identifying persistent neuroinflammation following TBI remain underexplored. Astrocyte‐derived small extracellular vesicles (AEVs) from plasma encapsulate inflammatory cytokines and complement proteins, offering a window into chronic neuroinflammatory processes linked to TBI and cognitive dysfunction. Previous studies demonstrate that complement proteins within AEVs predict mild cognitive impairment (MCI) conversion and are elevated in TBI patients years post‐injury. This study examines AEV cargo to determine whether inflammatory cytokines and complement proteins serve as biomarkers for cognitive decline in aging veterans with a history of TBI.

**Method:**

Participants of the Vietnam Era Twin Study of Aging (VETSA), a longitudinal cohort of male twins (mean age = 68), have extensive cognitive, laboratory, and genetic assessments. Plasma AEVs from biobanked VETSA3 samples were isolated using immunocapture of astrocyte‐specific marker GLAST (ACSA‐1) and characterized using nanoimaging. We quantified key inflammatory cytokines (IL‐1β, IL‐6, TNF‐α, IL‐10) and complement proteins (C4b and C3b) within AEV cargo using ultra‐sensitive SIMOA assays. Group differences between TBI‐exposed (N≈320) and non‐TBI (N≈680) participants were assessed using linear mixed‐effects models, accounting for twin clustering. Longitudinal mixed models were applied to evaluate the association of AEV protein cargo with cognitive decline, with MCI classification used as a secondary outcome. ROC analyses examined the predictive utility of AEV biomarkers for MCI diagnosis.

**Result:**

AEVs were successfully isolated and characterized, confirming the EV size, shape, and enrichment of astrocyte markers. Preliminary findings suggest significant elevations in C3b and IL‐6 in TBI‐exposed individuals compared to controls, with higher levels correlating with accelerated cognitive decline. ROC analyses indicate that a combined biomarker panel (IL‐6, TNF‐α, and C4b) improves the classification of MCI status (AUC > 0.7).

**Conclusion:**

These findings highlight the potential of AEV‐derived inflammatory and complement proteins as biomarkers of neuroinflammation and cognitive decline in TBI‐exposed aging populations. The persistence of inflammatory abnormalities in AEVs years after TBI suggests a chronic neuroinflammatory response that may underlie increased AD risk. Future work will examine interactions between inflammatory and neurodegenerative AEV biomarkers to refine risk prediction models for cognitive impairment in TBI populations.

